# Supplementary material for: Dietary and Activity Factors Influence Poor Sleep and the Sleep-Obesity Nexus among Children
Source: Int J Environ Res Public Health. 2019 May 20;16(10):1778. doi: 10.3390/ijerph16101778 (PMC6571639; doi:10.3390/ijerph16101778)
Supplement: Supplementary file 1 [file ijerph-16-01778-s001.pdf]

International Journal of
Environmental Research and Public Health

Supplementary Material

Dietary and Activity Factors Influence Poor Sleep and the Sleep-Obesity Nexus among Children

Bridget Morrissey, Steven Allender and Claudia Strugnell

Table S1. Data management: outline of variables and coding modifications.

	Variable	Original Coding	New Or Recoded Variable	Further recoding
Weight Status	Categorical BMI z-scores	**Weight Status: four categories:**<br>**-1** Thinness (BMI <-2SD)<br>**0** Normal weight (BMI ≥-2SD & ≤1SD)<br>**1** Overweight (BMI >1SD & <=2SD)<br>**2** Obesity (BMI >2SD)	**Weight Status three categories:**<br>**0** Normal weight (BMI <1SD)<br>**1** Overweight (BMI ≥1SD, <2SD)<br>**2** Obesity (BMI ≥2SD)	**Weight Status two categories:**<br>**0** Normal weight (BMI <1SD)<br>**1** Overweight (BMI ≥1SD)
Sleep Variables	Sleep duration	**There categories:**<br>**1** <9 hours (short)<br>**2** ≥9-<11 hours (sufficient)<br>**3** 11 or more hours (long)	**Two categories:**<br>**1** <9 hours (short)<br>**2** ≥9 hours (sufficient)	**Poor duration**<br>**0** YES (<9 hours)<br>**1** NO (≥91 hours)
	Sleep timing	**Bed time**<br>**1.** Early to bed ≤ 20:15<br>**2.** Late to bed ≥20:30<br>**Wake time**<br>**1.** Early to rise ≤7:00<br>**2.** Late to rise ≥7:15	**Sleep-wake timing categories**<br>**1** Early To Bed / Early To Rise<br>**2** Early To Bed / Late To Rise<br>**3** Late To Bed / Early To Rise<br>**4** Late To Bed / Late To Rise	**Poor bed time**<br>**0** YES (Late to bed ≥20:30)<br>**1** NO (Early to bed ≤ 20:15)<br>**Poor wake time**<br>**0.** YES (Early to rise ≤7:00)<br>**1.** NO (Late to rise ≥7:15)
	Sleep quality	**How well sleep**<br>**0** Very Good<br>**1** Fairly Good<br>**2** Average<br>**3** Fairly Bad<br>**4** Very Bad	**Three categories (reversed)**<br>**1** Bad/very bad<br>**2** Average<br>**3** Good	**Poor sleep quality**<br>**0** YES (bad/very bad)<br>**1** NO (good/average)

	**Sleep initiation**	**Problem falling asleep (>20 mins)**<br>**0** Never >20 (Good)<br>**1** Almost never > 20min<br>**2** Sometimes >20min<br>**3** Often >20min<br>**4** Almost always >20 min (bad)	**Three categories (reversed)**<br>**1** Bad/very bad<br>**2** Average<br>**3** Good	**Poor sleep initiation**<br>**0** YES (bad/very bad)<br>**1** NO (good/average)
	**Sleep maintenance**	How often wake up during the night<br>**0** Never (I don't)<br>**1** Almost never (every now and then)<br>**2** Sometimes (at least once a night)<br>**3** Often (3 times or more a night)<br>**4** I don't know (CONVERT TO MISSING)	**Three categories**<br>**1** Never<br>**2** Once or twice<br>3 Three or more	**Poor sleep maintenance**<br>**0** YES (≥3wake episodes)<br>**1** NO (<3 wake episodes)
	**Sleep score**	**Row total:**<br>Poor duration, poor bed time, poor wake time, poor sleep quality, poor sleep initiation, and poor sleep maintenance<br>(Range 0-6)	**Sleep score four categories**<br>**0** No sleep problem<br>**1** One sleep problem<br>**2** Two sleep problems<br>**3** Three sleep problems<br>**4** ≥Four sleep problems	**Sleep score three categories**<br>**0** ≤ One sleep problem<br>**1** Two sleep problems<br>**2** ≥Three sleep problems
**Dietary Behaviours**	**Fruit Guidelines**	**Reported average number of serves per day**<br>15 total options for number of serves<br>0.5 serve increments<br>Range from 0 serves to 7 serves per day	**Met recommended daily serves guidelines**<br>**0** No (< 2 serves)<br>**1** Yes (≥ 2 serves)	
	**Vegetable guidelines**	**Reported average number of serves per day**<br>15 total options for number of serves<br>0.5 serve increments<br>Range from 0 serves to 7 serves per day	**Met recommended daily serves guidelines**<br>***Boys aged 12years+:***<br>**0** No (<5.5 serves)<br>**1** Yes (≥ 5.5 serves)<br>***All girls and boys<12 years:***<br>**0** No (<5 serves)<br>**1** Yes (≥ 5 serves)	

	**Takeaway consumption**	**Average takeaway consumption as a meal**<br>**1** Every meal<br>**2** More than once a day<br>**3** Every day<br>**4** Almost every day<br>**5** 2-4 times a week<br>**6** Once a week<br>**7** Once a fortnight<br>**8** Rarely or never	**Four categories (reversed)**<br>**0** Once a week or less<br>**1** 2-4 times a week<br>**2** Every day/almost every day<br>**3** More than once a	**Two Categories**<br>**0** Once a week or less<br>**1** 2 times or more
	**Sugar sweetened beverage (SSB) consumption**	**Average SSB consumption**<br>**1** 3 or more times per day<br>**2** 2 times per day<br>**3** 1 time per day<br>**4** Almost every day<br>**5** Every second day<br>**6** Once a week<br>**7** Once a fortnight<br>**8** Rarely or never	**Four categories (reversed)**<br>**0** Once a week or less<br>**1** Almost every day/ every second day<br>**2** 1-2 times per day<br>**3** 3 or more times per day	
	**Sugar sweetened beverage before bed**	**How often drink SSB before bed**<br>**0** Never<br>**1** Almost never<br>**2** Sometimes<br>**3** Often<br>**4** Almost always	**Two categories**<br>**0** Never/sometimes<br>**1** Often/almost always	
	**Snack consumption**	**Average snack consumption**<br>**1** 3 or more times per day<br>**2** 2 times per day<br>**3** 1 time per day<br>**4** Almost every day<br>**5** Every second day<br>**6** Once a week<br>**7** Once a fortnight<br>**8** Rarely or never	**Four categories (reversed)**<br>**0** Once a week or less<br>**1** Almost every day/ every second day<br>**2** 1-2 times per day<br>**3** 3 or more times per day	**Two Categories**<br>**0** Once a day or less<br>**1** Twice or more per day
**Physical Activity Behaviours**	**Physical activity (PA) Guidelines**	**Meeting daily PA guidelines (separate for each day of the week)**<br>**0** Guidelines not met (<1 hour)	**Meeting PA guidelines (for all seven days)**<br>**0** < 7 days with ≥1 hour PA<br>**1** 7 days with ≥1 hour PA	**Meeting PA guidelines (for five of seven days)**<br>**0** < 5 days with ≥1 hour PA<br>**1** ≥5 days with ≥1 hour PA

		**1** Guidelines met (≥1 hour)		
	**Physically active 1 hour before bed**	**How often physically active 1 hour before bed**<br>**0** Never<br>**1** Almost never<br>**2** Sometimes<br>**3** Often<br>**4** Almost always	**Two categories**<br>**0** Never/sometimes<br>**1** Often/almost always	
**Screen Time Behaviours**	**Screen time (ST) guidelines**	**Meeting daily ST guidelines (separate for each day of the week)**<br>**0** Guidelines not met (>2 hours)<br>**1** Guidelines met (≤2 hours)	**Meeting ST guidelines (for all seven days)**<br>**0** < 7 days with≤2 hours ST<br>**1** 7 days with ≤2 hours ST	**Meeting ST guidelines (for all seven days)**<br>**0** < 5 days with ≤2 hours ST<br>**1** ≥5 days with ≤2 hours ST
	**Screen time 1 hour before bed**	**How use screen device 1 hour before bed**<br>**0** Never<br>**1** Almost never<br>**2** Sometimes<br>**3** Often<br>**4** Almost always	**Two categories**<br>**0** Never/sometimes<br>**1** Often/almost always	
	**Screen time in bed**	**How often use screen device while in bed**<br>**0** Never<br>**1** Almost never<br>**2** Sometimes<br>**3** Often<br>**4** Almost always	**Two categories**<br>**0** Never/sometimes<br>**1** Often/almost always	

© 2019 by the authors. Submitted for possible open access publication under the terms and conditions of the Creative Commons Attribution (CC BY) license (http://creativecommons.org/licenses/by/4.0/).
